# Comprehensive Impacts of Climate Change on Rice Production and Adaptive Strategies in China

**DOI:** 10.3389/fmicb.2022.926059

**Published:** 2022-06-30

**Authors:** Shah Saud, Depeng Wang, Shah Fahad, Hesham F. Alharby, Atif A. Bamagoos, Ali Mjrashi, Nadiyah M. Alabdallah, Saleha S. AlZahrani, Hamada AbdElgawad, Muhammad Adnan, R. Z. Sayyed, Shafaqat Ali, Shah Hassan

**Affiliations:** ^1^College of Life Sciences, Linyi University, Linyi, China; ^2^Hainan Key Laboratory for Sustainable Utilization of Tropical Bioresource, College of Tropical Crops, Hainan University, Haikou, China; ^3^Department of Agronomy, University of Haripur, Haripur, Pakistan; ^4^Department of Biological Sciences, Faculty of Science, King Abdulaziz University, Jeddah, Saudi Arabia; ^5^Department of Biology, College of Science, Taif University, Taif, Saudi Arabia; ^6^Department of Biology, College of Science, Imam Abdulrahman Bin Faisal University, Dammam, Saudi Arabia; ^7^Botany and Microbiology Department, Faculty of Science, Beni-Suef University, Beni Suef, Egypt; ^8^Department of Agriculture, The University of Swabi, Swabi, Pakistan; ^9^Department of Microbiology, PSGVP Mandal’s S. I. Patil Arts, G. B. Patel Science and S. T. K. V. Sangh Commerce College, Shahada, India; ^10^Department of Environmental Science and Engineering, Government College University, Faisalabad, Pakistan; ^11^Department of Agricultural Extension Education and Communication, The University of Agriculture, Peshawar, Pakistan

**Keywords:** food security, global warming, northern boundary, rice planting system, growth stage, grain yield

## Abstract

The rice production system is one of the most climate change sensitive agro-ecosystems. This paper reviews the effects of current and future climate change on rice production in China. In recent decades, thermal resources have increased during the rice growing season, while solar radiation resources have decreased, and precipitation heterogeneity has increased. The increasing frequency of high-temperature stress, heavy rainfall, drought, and flood disasters may reduce the utilization efficiency of hydrothermal resources. Climate change, thus far, has resulted in a significant northward shift in the potential planting boundaries of single- and double-cropping rice production systems, which negatively affects the growth duration of single-, early-, and late-cropping rice. Studies based on statistical and process-based crop models show that climate change has affected rice production in China. The effects of climate change on the yield of single rice (SR), early rice (ER), and late rice (LR) were significant; however, the results of different methods and different rice growing areas were different to some extent. The trend of a longer growth period and higher yield of rice reflects the ability of China’s rice production system to adapt to climate change by adjusting planting regionalization and improving varieties and cultivation techniques. The results of the impact assessment under different climate scenarios indicated that the rice growth period would shorten and yield would decrease in the future. This means that climate change will seriously affect China’s rice production and food security. Further research requires a deeper understanding of abiotic stress physiology and its integration into ecophysiological models to reduce the uncertainty of impact assessment and expand the systematicness of impact assessment.

## Introduction

According to the Fifth Assessment Report of the Intergovernmental Panel on Climate Change (IPCC_AR5), the global average surface temperature increased by approximately 0.85°C from 1880 to 2012, and the global surface temperature has risen continuously in the past 10-year historical period. Changes in the climate system have had a general impact on global food production, and the risk of climate change severely affecting crop yields in the future may also increase (Pachauri and Meyer, 2014).

Rice is the main ration crop in China, and more than 65% of the population in China eat rice as their main food source (Maclean et al., 2002; Chen et al., 2017d). According to statistics, from 2012 to 2016, the average annual sowing area of rice in China was 3.023 × 10^7^ hm^2^, which accounts for 26.9% of the average sown area of rice globally (11.245 × 10^7^ hm^2^). The average annual rice yield was 2.059 × 10^8^ t, which is 33.9% of the annual global rice yield (6.072 × 10^8^ t) (Alexandratos and Bruinsma, 2012). Rice yield has doubled in all counties and cities in China in the last 30 years (IPCC, 2013), which may be related to climate changes such as temperature and solar radiation (Bongaarts, 2019). Therefore, it is more important than ever to scientifically assess the impact of climate change on rice production and formulate effective coping strategies to provide theoretical support for overcoming rice yield shortages.

China is the largest producer, consumer and importer of rice in the world, and more than 80% of the Chinese population relies on rice as a staple food. A high yield of rice is the cornerstone of food security in China and even the world (Chen et al., 2017d). According to the China Climate Change Blue Book released in 2018 by the China Weather Administration. China is sensitive to, and severely affected by global climate change, and the annual average surface temperature in China increased by 1.6°C from 1951 to 2017. China’s warming rate is not only higher than the global average over the same period but it has also been subject to more frequent extreme weather phenomena, such as high and low temperature damage; therefore, the impact of global warming on China’s rice production could be more prominent than in other countries (Peng et al., 2004; Lobell et al., 2011; Tao and Zhang, 2013; Zhao et al., 2017a). In addition, China’s rice growing area is vast, from the Yunnan-Guizhou Plateau to the eastern coastal delta, and from Heilongjiang Mohe to Hainan, there are significant differences in the temperature of the rice growing season in different regions, and the impact of climate warming on China’s rice production will also have significant temporal and spatial differences (Tao et al., 2013; Chen et al., 2017c). Furthermore, China has a variety of rice-growing patterns and systems, including southern dual cropping rice, (SD) Yangtze River basin (YRB), medium-cultivation rice,(MR) and northern single cropping rice, (N) covering almost all rice cropping patterns in the world. The responses of rice growing seasons and rice yield to climate warming in different rice-growing patterns will also have their own characteristics (Chen et al., 2017c; Guo et al., 2020; Zhang et al., 2020).

A comprehensive analysis of the global warming effects is of seminal importance for the theory and technological innovation of adaptive crop cultivation under climate change. To date, there have been a large number of studies on the impact of climate warming on crop production, basically clarifying the response characteristics of global food production (Lobell et al., 2011; Zhang, 2014; Liu et al., 2016). However, most of the existing research is based on model analysis and historical data mining, and there are insufficient data to summarize field experimental research and long-term observations. There is still significant uncertainty about the impact of climate change on specific countries (planting regions) and specific seasonal crops (Zhao et al., 2017b; Liu et al., 2018). In recent years, experimental research on the response and adaptation of crop growth to climate warming has received increasing attention, and long-term field observation data have increased year by year. These experiments and observations have not only improved the understanding of climate warming and crop response by academia and the public but also provided a wealth of empirical data for comprehensive analysis. Based on the regional characteristics of Chinese perennial rice, the response characteristics and adaptation trends of rice fertility, yield and quality to temperature rise in a typical Chinese rice planting system are comprehensively analyzed. According to the researcher’s multiple planting experiments and long-term observations. This study aims to provide a theoretical basis and technical suggestions for the effect of climate warming on increasing green rice production.

## Climate Warming in China’s Main Grain-Producing Areas

According to historical meteorological monitoring, global warming presents significant regional, seasonal and diurnal variations (IPCC, 2013). The primary trend is that the temperature rise in high latitude areas is significantly higher than that in low latitude areas, and the northern boundary of crop cultivation will expand northward and the area will increase. The warming span in summer and autumn was significantly lower than that in winter and spring. The temperature rise during the daytime was significantly lower than that at night, and the temperature difference narrowed, which may be unfavorable to the formation of crop yield and quality. Due to the differences in background temperature of crop growing seasons in different regions and seasons, as well as the difference in temperature increases in corresponding regions and seasons, there are obvious spatiotemporal characteristics of the impact of climate warming on crop production (Lobell et al., 2011). Therefore, mastering the spatiotemporal characteristics and trends of temperature changes in specific countries (planting regions) will be helpful to fully understand the comprehensive response of crop production to climate warming.

A large number of existing meteorological monitoring data and model prediction analysis results show that the trend of climate warming in China over the past few decades has been significantly higher than that observed from global monitoring data, according to the China Meteorological Station from 1970 to 2017 (Chen et al., 2020). The spatiotemporal differences in temperature rise in the three major grain-producing regions of Northeast China and the Yangtze River Delta (YRD) are significant. Compared to the 1970s, the daily minimum temperature in the 2010 harvest season increased by 1.39 and 0.70°C in Northeast China, by 1.35 and 0.86°C in North China and by 1.28 and 1°C in the YRD region. During the same period, the average temperature in winter, spring, summer and autumn in Northeast China increased by 1.18 and 0.89°C. The average temperature in North China increased by 1.31 and 0.67°C, and that in the YRD it increased by 1.28 and 0.99°C, which is similar to the overall trend of global warming.

At the same time, the warming trend and precipitation days in the growing season of major grain-producing areas in China have obvious regional changes; that is, precipitation in the western part of China has an increasing trend, while precipitation in the eastern part of China has a decreasing trend. In addition, the frequency of high-intensity precipitation has increased, particularly in Southeast China, where the total precipitation showed a slight downward trend; however, the frequency of heavy rain and storms showed a significant upward trend (Jiang Y. et al., 2017). Overall, there was no significant change in total precipitation in the crop growing season, but the number of days of precipitation significantly decreased, the intensity of daily precipitation increased significantly, and the problem of water-heat mismatch in the crop growing season became more prominent.

Under the influence of temperature and precipitation changes, the frequency of seasonal drought and extreme temperature changes also showed an increasing trend. The occurrence of summer high-temperature and drought disasters is increasing in most regions of China. Vicente-Serrano et al. (2010) proposed the standardized precipitation-evapotranspiration index (SPEI), which represents the degree of deviation of dry and wet conditions in an area when compared to a regular year by determining the difference between the SPEI, which can be used to analyze the trend of drought evolution. Taking the SPEI during the crop cultivation period from 1970 to 2017 in China’s main grain-producing areas as an example, the SPEI value increased by 0.36 in the spring cultivation season in Northeast China, decreased by 0.59 in the summer cultivation season in the North region of China, and increased by 0.86 in the autumn cultivation season in the YRD. There were obvious regional differences between Northeast China and the YRD. There are obvious new trends in warm–dry, wet–heat, and dry–heat. At the same time, climate warming has led to the frequency of extreme disasters, and its spatial distribution has also shown significant differences. Compared with Northeast, Northwest and North China, the climate change range in southern China is relatively small, but there is more subtropical high pressure in summer, which has led to an increase in extreme high temperature events in the south. In addition, despite the warming trend in the northwest region, extreme low temperature events have increased significantly since the 1980s (Wang et al., 2012). Overall, there are more extreme climate events in the Northwest region and the middle and lower reaches of the YR, while there are fewer extreme climate events in the Northeast region and the middle and upper reaches of the YRB (Ju et al., 2013b; Xie et al., 2018; Chandio et al., 2020).

## The Influence of Climate Change on Rice Production in China

Rice production is a complex natural-social system in which long-term changes in rice yield are mixed with climate change and anthropogenic signals. In general, the measured yield per unit area of single cropping rice and early and late rice increased by 0.69 (0.37–1.07) t hm^–2^ per decade from 1980ά to 2010 (Table 1). Due to the influence of climatic factors, climate change has had a negative impact on rice yields in China in recent decades. The evaluation based on the rice growth model (Table 1) showed that the change in the mean climate between 1980ά and 2010 reduced the rice yield per unit area by 0.25 (0.01–0.56) t hm^–2^ 10 yr^–1^. From 1961ά to 2010, the rice yield per unit area decreased by 12.0% (11.5–12.4%) (Liu et al., 2012, 2013). The interannual fluctuation of rice yield can be reduced by planting varieties with strong stress resistance or improving cultivation and management measures (Osborne and Wheeler, 2013). The positive effects of variety improvement and rational fertilization on rice yield even exceeded the negative effects of climate change (Yu et al., 2012; Li et al., 2014; Xiong et al., 2014). In conclusion, although climate change has seriously restricted the growth of rice yield, China’s rice production system has actively dealt with these adverse effects in an appropriate manner, and the rice yield has steadily increased. However, climate change will continue to severely limit the contribution of technological advances to food production in the future (Wang et al., 2009; Zhang, 2014; Xie et al., 2018) and increase the difficulty of agricultural technological innovation.

**TABLE 1:** Impact of climate change on rice grain yield in China.

Change trend						
Rice system	Region	Period	Statistical model^a^ (t hm^–2^ 10 yr^–1^)	Crop model^b^ (t hm^–2^ 10 yr^–1^)	Method	References
SR	4 stations of China	1981–2009	0.87	–0.45	Rice grow	Liu et al., 2012
SR	NE China	1980–2010	1.08	–0.01	ORYZA rice	Zhang et al., 2014
SR	N China	1980–2010	0.59	–0.32	ORYZA rice	Zhang et al., 2014
ER	3 stations of DR experiment	1981–2009	0.38	–0.09	Rice grow	Liu et al., 2013
LR	3 stations of DR experiment	1981–2009	0.52	–0.11	Rice grow	Liu et al., 2013
SR and DR	Eastern China	1980–2010	0.62	–0.57	ORYZA rice	Zhang et al., 2014
SR and DR	Central China	1980–2010	0.64	–0.28	ORYZA rice	Zhang et al., 2014
SR and DR	Southwest China	1980–2010	0.87	–0.27	ORYZA rice	Zhang et al., 2014
DR	Southern China	1980–2010	0.76	–0.18	ORYZA rice	Zhang et al., 2014
ά	Average	1980–2010	0.68	–0.26	ά	ά
Rice	China	1961–2010	ά	–11.6%	CERES-rice	Yang et al., 2014
Rice	China	1961–2010	ά	–12.5% (*–4.3%*)	CERES-rice	Xiong et al., 2012
ά	Average	1961–2010	ά	–12.1%	ά	ά
Rice	China	1961–2010	ά	(*2.0%*)	EPIC, DSSAT	Jones and Jones, 2003; Xiong et al., 2014
Rice	China	1981–2009	ά	(*4.5%*)	GAEZ	Yu et al., 2012
SR	China	1981–2009	ά	(*3.5%*)	GAEZ	Yu et al., 2012
ER	DR region	1981–2009	ά	(*4.9%*)	GAEZ	Yu et al., 2012
LR	DR region	1981–2009	ά	(*7.9%*)	GAEZ	Yu et al., 2012
SR	NE China	1981–2009	1.02–3.28%	ά	Panel model	Tao et al., 2013
SR	Middle and lower reaches of YR	1981–2009	–9.69 to –7.15%	ά	Panel model	Tao et al., 2013
ER	Middle and lower reaches of YR	1981–2009	–0.59 to 2.40%	ά	Panel model	Tao et al., 2013
LR	Middle and lower reaches of YR	1981–2009	8.38–9.56%	ά	Panel model	Tao et al., 2013
Rice	Southern China	Elevated temperature 1δ	–3.48 to –2.52%	ά	Economy-Climate model	Li et al., 2020
DR	Southern China	1980–2008	–0.17% yr^–1^	ά	Statistical model	Wang et al., 2014
SR	NE China	1980–2008	0.59% yr^–1^	ά	Statistical model	Wang et al., 2014
SR	Yunnan-Guizhou Plateau	1980–2008	0.34% yr^–1^	ά	Statistical model	Wang et al., 2014
SR	Sichuan Basin	1980–2008	–0.29% yr^–1^	ά	Statistical model	Wang et al., 2014

*A Statistical model column is based on the analysis results of historical measured rice yield data by statistical model. The Statistical Model valves from 0.87 to 0.68 are the change trend of measured rice yield over time, and the other values are the response of measured rice yield to climate change.*

*^a^The crop model column is based on the rice growth model, and the variety and management parameters are set as the change trend or change percentage of the simulated yield with fixed value (ά) indicate measured yield per unit area of SR, LR, DR. The values in italic are the simulation results considering the increase of CO_2_ concentration, and the other values are the simulation results with constant CO_2_ concentration.*

*SR, Single rice; DR, Double rice; ER, Early rice; LR, late rice; NE, Northeast China; N, North; YR, Yangtze River.*

*Represent for the simulations with elevated CO_2_ concentration.*

The influence of climate change factors on rice production in China is related to the region and the type of rice cultivation. The results based on statistical and growth models (Table 1) showed that under the influence of long-term climate change, rice production in northern, eastern and central China (single-season rice in the middle and lower reaches of the YR) and in southwestern China (single-season rice in the Sichuan Basin) and DR in southern China declined significantly, while SR yields increased in the middle and lower reaches of the YR, northeastern China and the Yunnan-Guizhou Plateau (YGP). Extreme weather is another important reason for rice production reduction, and its impact on rice yield may be greater than long-term changes in climate factors and interannual fluctuations (Espe et al., 2017; Wang et al., 2018). In the past 30 years, extreme temperature stress in China has led to a yield loss of approximately 6.1% of irrigated rice in China, and the yield losses of single cropping rice in the Sichuan Basin, single cropping rice in the middle and lower reaches of the YR, and of early rice have increased significantly in southern China (Alexandratos and Bruinsma, 2012; Wang et al., 2016). In addition, reasonable allocation of climate resources helps improve rice yield and utilization efficiency of light and temperature resources (Deng et al., 2015), while inappropriate allocation of resources can lead to serious yield losses (Tao et al., 2016). Other studies have shown that aerosol concentration affects the ratio of incident solar radiation and scattered radiation, and severe air pollution can have adverse effects on rice yield (Tie et al., 2016; Zhang et al., 2017). Contrary to the adverse atmospheric environment, the increase in atmospheric CO_2_ concentration is beneficial for increasing rice yield (Xiong et al., 2012), and the response of late rice yield to the increase in atmospheric CO_2_ concentration is greater than the yield loss of early rice in response to climate change (Yu et al., 2012; Xiong et al., 2014). The effect of increased CO_2_ concentration largely increased the yield, or even almost compensated, for the yield decrease caused by climate change.

### Background Temperature and Warming Trend for Typical Chinese Rice Cropping Systems

**FIGURE 1:**
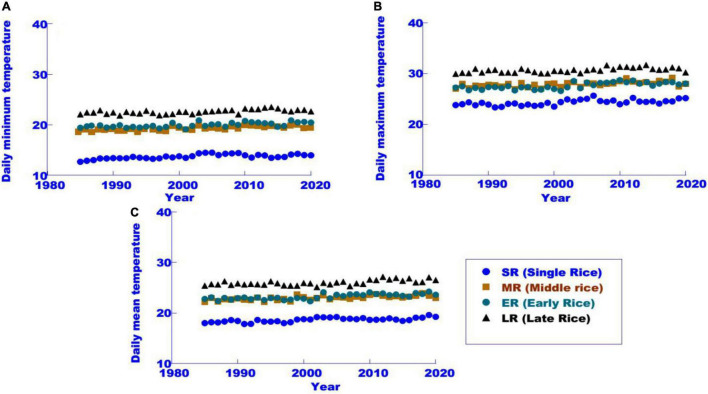
Daily minimum temperature **(A)**, maximum temperature **(B)**, and average temperature **(C)** of the rice growing season in the main rice-growing regions of China during 1980–2020 (modified from Deng et al., 2019).

Typical rice cropping systems in China include a single cropping system in the northern region represented by northeast China, a medium cropping system in the middle and lower reaches of the YR, and a double cropping system in south China. The climatic background of the three rice cropping systems is significantly different. Based on annual temperature changes from 1980 to 2015 (Chen et al., 2020), the maximum and minimum daily temperatures were 18.8, 24.4, and 13.6°C, respectively, and the warming ranges were 0.31, 0.29, and 0.36°C⋅10^–1^a, respectively. The daily mean, maximum and minimum temperatures were 23.0, 28.0, and 19.3°C, respectively, and the amplitudes of temperature were 0.34, 0.39, and 0.32°C⋅10^–1^a. The corresponding early rice (ER) background temperatures were 23.3, 27.7, and 20.0°C, with slopes of 0.28, 0.29, and 0.29°C⋅10^–1^a, respectively. The corresponding background temperatures of late rice (LR) were 26.0, 30.7, and 22.6°C, respectively, and the amplitudes of the air temperature rise were 0.25, 0.26, and 0.25°C 10^–1^a. In terms of the overall trend (Figure 1), the temperature of the rice growing season increased significantly under the three rice cropping systems. The background temperature of middle rice (MR) and ER was similar in South China. The postanthesis background temperature of first-season rice in Northeast China was similar to that of DR and LR and was significantly lower than that of MR and ER. Due to the significant differences in background temperature and warming amplitude among different rice cropping systems, the effects of climate warming on different rice cropping systems also have significant differences.

### Response Characteristics of the Growing Season of Rice and Elevation of Yield at Temperature

Currently, researchers have conducted a large number of field warming experiments in major rice-growing areas in China. For example, from 2016 to 2020, a warming experiment was conducted in Harbin, Heilongjiang Province, a single-cropping rice region in northern China. A nighttime warming experiment was conducted in Nanjing, Jiangsu Province, on medium-cropping rice in 2008 within flooding and drought conditions. From 2007 to 2011, nighttime warming experiments were carried out in Nanchang, Jiangxi, on dual-cropping rice in southern China which clarified the response and adaptation of different rice growing periods and productivity in different rice cropping systems (Dong et al., 2011; Chen et al., 2017a; Rehmani et al., 2021). The results showed that the growth period from sowing to heading and flowering of rice was significantly shortened when the temperature increased by 1.5°C, and the grain filling period of Heilongjiang and Nanchang double-season rice was even prolonged. In general, although the temperature rise significantly shortened the whole growth period of rice, it should be noted that the temperature increase can shorten the reproductive period of rice before flowering; however, the reproductive period after flowering, in general, does not change nor is extended. Similar changes in crop phenology have also been confirmed in long-term field observations (Tao et al., 2013).

Years of field warming experiments also found significant differences in the response characteristics of rice productivity under different rice cropping systems (Deng et al., 2017). When the temperature increased by 1.5°C, the biological and grain yields of single cropping rice in Harbin increased significantly, the biological and grain yields of Nanjing MR decreased, while ER decreased and LR increased in Nanchang DCR. Based on the current rice planting layout and the response differences of the three rice cropping systems. Further analysis of the results of the regional combined multiyear point sowing experiment showed that rice yield was mainly affected by background temperature after flowering. Increasing the temperature can significantly increase the leaf area of rice, which is beneficial for the accumulation of dry matter and yield formation of rice (Yang et al., 2019). Therefore, in the regions with a high post anthracite background temperature, such as MR in Nanjing and ER in Nanchang DCR, when the temperature increased by 1.5°C, panicle differentiation and flowering fertilization of rice were more susceptible to heat damage and the seed setting rate, and the decrease in grain number per panicle led to yield reduction. However, if the background temperature is low, such as for the LR of single cropping rice in Harbin and DR in Nanchang, a temperature increase of 1.5°C can promote an increase in the effective panicle and grain number per panicle, which is conducive to increasing yield (Chen et al., 2017b).

**FIGURE 2:**
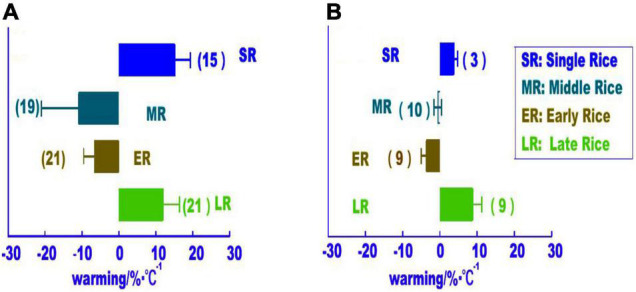
Effects of climate warming on rice yield per unit area in different rice cropping systems. **(A)** Observational data from long-term field trials; **(B)** analysis based on provincial statistical data. Error bars indicate standard errors (modified from Lv et al., 2018).

A similar warming effect was observed based on long-term site-based testing of three rice cropping systems and analysis of provincial statistical data (Figure 2). Long-term site test results showed that with an increase in the average temperature of the rice-growing season by 1.0°C rice yield per unit area under single cropping increased by 15.3% in northeast China, while rice yield per unit area decreased by 10.9% in moderately flooded and arid cropping areas, and earlier rice yield per unit area increased by 6.7 and 12.1% on average in southern China under single and late cropping seasons, respectively. The provincial statistics showed similar results when the average temperature of the rice growing season increased by 1.0°C, the average yield of the SR season in Northeast China increased by 3.8%, and that of the DR in flood and drought conditions increased by 0.6%. In southern China, the rate was 3.7% for ER and 8.9% for LR. In general, the rice yields of the three rice cropping systems in China showed both an increasing and decreasing trend due to climate warming but generally remained stable (Jiang et al., 2021).

### Variation in Rice Planting Regions and Contribution to the Portion of Total Rice Yield

**FIGURE 3:**
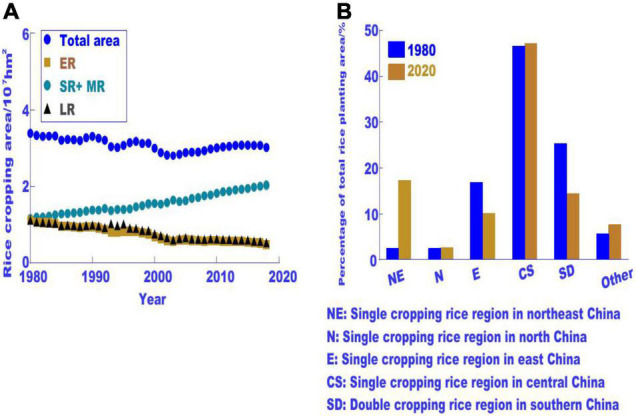
Changes in rice planting area in China over the past 40 years. **(A)** Changes in rice planting area in different rice cropping types; **(B)** differences in the percentage of rice planting area in the total rice area of China in different rice growing regions (modified from Lv et al., 2018).

With the increase in temperature and socioeconomic development, the area under rice cultivation in China has changed significantly, and the contribution of rice yield to the total rice production in China under different regional rice cropping systems has also changed significantly (NBSC, 2019). The area under rice cultivation, especially for DCR (double cropping rice), decreased significantly in the south, while the area under rice cultivation in the north increased rapidly, from 5% in 1980 to 20% in 2018 (Figure 3). Compared with 1950, the rice planting area in Guangdong Province decreased by more than 60% to 1.8 × 106 hm^2^ in 2015 in Heilongjiang Province, and the rice planting area increased by more than 30 times, reaching 4.0 × 106 hm^2^. This is related not only to the difference in economic development between the north and south but also to the significant rise in temperature in the northeast, which results in a cumulative effect of climate warming and economic development.

The yield contribution rate of different rice cropping systems also changed significantly. In southern China, the area planted with double cropping rice decreased significantly, while the area planted with medium rice increased rapidly. The contribution of medium rice to the total rice yield in China will gradually increase (Figure 3). In 1980, the area and yield of DCR in southern China accounted for 65.8 and 61.4% of the total rice production in China, respectively. In 2020, the proportion of double cropping rice decreased to 33.3 and 28.3%, respectively, with significant changes (Chen et al., 2017c). With the advancement of the national economy, the area of paddy fields in South China has decreased, and the regional advantages of different rice-producing areas have changed significantly. Due to rice regionalization and changes in rice planting systems due to climate warming, such as the moderate expansion of rice in the future, which is the most sensitive to temperature change, it will further aggravate the negative impact of rising temperature on China’s rice production and endanger national food security.

### Characteristics of Rice Quality Response to Climate Change

With the advancement of human requirements for improved quality of life, the demand for high-quality rice is also increasing. Temperature changes have a significant impact on rice quality, with amylose and protein contents being the most sensitive parameters after temperature increases (Lin et al., 2010; Morita et al., 2016; Zhao et al., 2017a; Dou et al., 2018; Siddik et al., 2019). An increase in temperature significantly reduces the amylose content, increases the average grain size of starch and significantly increases the protein content (Liu J. C. et al., 2017). Such changes in starch and protein content can make rice fragile during processing and milling, and impact the appearance quality, significantly reduce the brown rice milled rate, and increase the chalkiness of rice (Lin et al., 2010; Siddik et al., 2019). The nutritional quality of rice is also sensitive to temperature increases, causing significant changes in nutritional components such as starch, storage protein and fatty acids in rice grains. Temperature increases can also increase the peak viscosity, hot slurry viscosity, final viscosity, disintegration value and gelatinization temperature of rice starch and reduce the flavor quality (Dou et al., 2018).

In previous studies, it was found that rising temperature in the growth period can affect rice grain formation, and that climate warming changes the growth process of rice, leading to the occurrence of extreme temperature in the growth period, and its occurrence stage and duration also change (Dong et al., 2011; Tao and Zhang, 2013; Zhang et al., 2014; Deng et al., 2017; Zhou et al., 2021). Through an artificial climate chamber experiment (Siddik et al., 2019), it was found that the second week after heading was a critical period in which temperature affected the formation of rice quality. The gelatinization temperature and protein content increased significantly, while the amylose content decreased. Rehmani et al. (2014) and Chen et al. (2016) showed in a field experiment with open heating that although postflowering heating led to a deterioration in the appearance quality of early and late rice, it improved the processing quality of LR and improved the nutritional quality of ER and LR to a certain extent, such as the improvement of protein content. In general, the effect of extreme temperature on rice quality is mainly caused by irreversible changes in grain filling and material accumulation in the critical period of grain formation. When the average daily temperature exceeds the critical threshold (>33°C) during rice filling, the yield and quality of rice will be adversely affected (Siddik et al., 2019). Extreme low temperatures at the rice filling stage also reduce rice quality (Song et al., 2011). In general, the effects of climate warming on rice quality are more harmful than beneficial, so emphasis should focus on developing and popularizing rice cultivation measures to cope with climate change.

## Effects of Future Climate Change on Rice Production

### Positive Impact on Rice Production

In the 1930s, 1950s, and 1970s, the daily average temperature of the rice growing season in China was 0.8–2.7°C, 1.7–3.4°C, and 2.3–4.1°C, respectively, higher than that in the first decade of the twenty-first century (Lv et al., 2018). The potential boundary between double and triple harvests in China will continue to move northward (Ju et al., 2013a; Tian et al., 2014), and the potential share of the triple-growing system in the total area of the planting system will increase by the end of the twenty-first century, reaching a maximum of 75.0% (Yang et al., 2015). The potential planting boundary of single- and double-cropping rice will continue to move northward in the future. Compared with 1961–1990, the expandable planting area of single- and double-cropping rice in China in the 2080s will be approximately 5.0 × 105 hm^2^ and 6.2 × 106 hm^2^, respectively (Xiong et al., 2009). The increase in heat resources extends the potential growing season of crops and significantly increases the growing season elasticity of rice (Ohta and Kimura, 2007; Tian et al., 2014), which is conducive to the flexible formulation of climate protection strategies for rice production.

### Adverse Impact on Rice Production

According to the IPCC Fifth Assessment Report, adverse effects of climate change and extreme climate events on crop yields are common (Pachauri and Meyer, 2014). If the temperature increases by 1–3°C in the future, the probability of shortening the rice growth period in China is 100% (Tao et al., 2008). When the temperature increases 1.5 and 2.0°C, the growth period of DCR in China will be shortened by 4–8% and 6–10%, respectively, and the growth period of SCR will be shortened by approximately 2% (Chen et al., 2018). A study combining grid crop models, single point crop models, statistical models and observational experiments showed that a temperature increase of 1% could lead to an average 3.2% decrease in global rice yield (Zhao et al., 2017b). By the end of the twenty-first century, sustained temperature increases are expected to reduce global rice yields by 3.4–10.9% (Table 2). The range of rice yield in China due to future climate change is –40.2 to 6.2%, with an average yield reduction of 10.6%, and the spatial difference is obvious (Table 2). If the impact of increased CO_2_ concentration on yield is considered, it has a certain compensation effect on the production reduction caused by climate change (Table 2). However, such compensation cannot offset the adverse effects of high temperature increases in some scenarios and regions or reduce interannual variability in rice yield (Tao et al., 2008; Xiong et al., 2009). In addition, the increase in precipitation and temperature variability may lead to an increase in frequency and reduction in low-yield years (Yao et al., 2007; Xiong et al., 2009).

**TABLE 2:** Impact of future climate change on rice grain yield.

Change trend*^c^*											
Rice system	Region	Period	Baseline	Climate scenario^a^	Climate model^b^	Crop model	Climate change (%)	CO_2_ effect (%)	Adaptation (%)	CO_2_ effect + Adaptation	References
SR	Eastern China	2020s, 2050s, 2080s	1961–1990	A1F1,B1	5 GCMs	MCWLA-Rice	–15.9 (–29.5 to –3.8)	8.5 (0.7–13.3)	–	–	Tao et al., 2013
SR and DR	Middle and lower reaches of Yangtze River	2021–2050	1961–1990	A2, B2	PRECIS RCM	ORYZA2000	–15.2	–5.6	–	–	Ding et al., 2019
ER and DR	Southern China	2071–2090	1961–1990	B2	PRECIS RCM	CERES-RICE	–3.9 (–7.0 to –0.21	8.7 (5.0–20.1)	–	–	Yao et al., 2007
SR and DR	Six station of China	2001–2100	1961–1990	ETI°C, 2°C, 3°C	5 GCMs	CERES-RICE	–20.5 (–40.3 to –6.2)	–4.9 (–19.4 to 0.19)	–	–	Tao et al., 2008
SR and DR	China	2020s, 2050s, 2080s	1961–1990	A2, B2	PRECIS RCM	CERES-RICE	–10.9 (–26.3 to 6.4)	3.6 (–5.7 to 15.9)	–	–	Xiong et al., 2009
ER and LR	Double Rice Region	–	1961–1990	A (ET1.7°C)	DKRZ OPYC(LSG)	MCWLA-**Rice**	–15.3 (–19.0 to –11.3)	–	8.8 (–7.1 to 23.2)	–	Li et al., 2020
Rice	China	2020s, 2030s, 2040, 2050s	2009	A2, B2	PRECIS RCM	CERES-RICE	–	10.6 (6.1–18.1)	–	15.9 (11.01–21.01)	Ye et al., 2013
E and DR	China	2011–2050	2000–2009	A2, B2	PRECIS RCM	Agro __C	–3.4	20.09	3.4	28.7	Yu et al., 2014
E and DR	China	2030s, 2050s, 2070s	2000s	RCP4.5	17 GCMs	CERES–RICE	–	–0.09 (–11.0 to 12.0)	4.9 (1.1–11.0)	–	Lv et al., 2018
ER and DR	China	2106–2115	200–2015	ET1.5°C, 2.0°C	4 GCMs	MCWLA-Rice	–0.9 (–0.8, 2.5)	6.9 (42.1, 9.5)	–	–	Chen et al., 2018
–	–	–	–	–	–		–10.8	5.4	5.7	22.4	
Rice	World	2070–2100	1981–2010	RCP2.6, 4.5, 6.0, 8.5	11–22 ESMs	7 global grid –based models	–3.4, –5.6, –6.9, –10.9	–	–	–	Zhao et al., 2017a

*SR, Single rice; DR, Double rice; ER, Early rice; LR, late rice.*

*(a) ET stands for elevated temperature; (b) 5 GCMs are HadCM3, PCM, CGCM2, CSI R02 and ECHAM4; 4 GCMs are CAM4, ECHAM6, MI ROCS and NorESM1; (c) Values in the climate change column only in connection with climate change, values in the CO_2_ effect column also take into account the increased CO_2_ concentration, values in the adaptation column also take into account the adaptation measures and values in the CO_2_ effect + adaptation column also take into account both the effects of a increased CO_2_ concentration and adaptation measures.*

The areas with the most obvious decrease in rice yield and increase in rice instability are the Sichuan Basin (SB), YRB, and Huang-Huai-Hai Plain (HHHP), which may become highly sensitive areas for rice due to future climate change (Xiong et al., 2009). Studies have also shown that the adverse effects of climate change on rice yield can be effectively mitigated if appropriate coping strategies are adopted (Table 2). In the future, there will be a need to conduct research on the measures to cope with climate change in rice production from the aspects of cultivating varieties with strong stress resistance and high utilization of CO_2_ concentration, optimization of cultivation management and anti-stress cultivation techniques, and adaptation to strengthen sowing date and planting area. In particular, a growing number of impact assessments have focused on changes in extreme weather events and their potential impact on rice production (Zhang et al., 2017, Zhang et al., 2018; Chen et al., 2018; Huang et al., 2018). From the 2000s to the 2050s, the area affected by extreme high-temperature stress in the global reproductive growing season of rice will increase from 8 to 27% (Alexandratos and Bruinsma, 2012; Gourdji et al., 2013). The probability, intensity and area of rice production subjected to high temperature stress in China will also increase, which may offset the positive effect of increased heat resources and reduced damage caused by low temperature (Tao et al., 2013; Wang et al., 2014; Zhang et al., 2016). When temperature rises by 1.5 and 2.0°C, rice yield in China may decrease by 2 and 5% under heat stress, respectively (Chen et al., 2018). The Sichuan Basin and the middle and lower reaches of the YR may become areas of high temperature heat damage, while Northeast China, the Yunnan-Guizhou Plateau YGP and East China are more at risk of severe low temperature damage than other regions (Wang et al., 2014; Zhang et al., 2017). In the future, increased precipitation variability may lead to an increased frequency of seasonal drought and heavy rain (Cai et al., 2018). In the eastern province of Jiangsu and other regions, extreme precipitation events may have a more significant impact on rice yield than extreme temperature events (Huang et al., 2018). In addition, rising temperatures will lead to an overall increase in evapotranspiration from reference crops, and southwestern China will experience an aridification process with a significant decrease in the wetness index (Tian et al., 2014).

## Problems and Prospects

In recent years, a large number of studies have been carried out on the comprehensive impact of climate warming on crop production and its countermeasures. The trend of climate warming and the response characteristics of the crop growth period and productivity have been clarified, and some adaptive planting technologies and coping strategies have been developed. However, there are still great uncertainties in understanding the response and adaptation of specific regions and crops to future climate warming in their growing seasons, and there is still a lack of holistic coping techniques in adaptive production and coping strategies. Therefore, systematic theoretical research and innovation of key technologies and models of regional adaptation are urgently needed.

### Strengthen Research on Climate Change Impact Mechanisms and Their Application in Impact Assessment

First, in theoretical research on crop response and adaptation to climate warming, the integration of field empirical research and regional model analysis should be further improved upon in the future. Existing studies mostly focus on model analysis and historical data mining, and few empirical studies in the field mainly focus on the single factor of temperature change. However, climate warming is not a single mean temperature change but also includes extreme weather and precipitation changes, as well as the accompanying changes in atmospheric composition, especially in atmospheric CO_2_ and near-surface O_3_. Therefore, the impact of climate warming on crop production is a combination of multiple factors, and comprehensive field demonstration and multifactor model mining are needed to clarify the comprehensive impact of climate warming and even climate change on crop production and reduce the uncertainty of future understanding.

### Reducing Uncertainty in Climate Change Impact Assessments

Second, there is an urgent need for innovation in research content, methods and means. Existing studies mostly focus on crop growth period and productivity, but research on crop product quality and safety, which is increasingly a concern of society, is still very unclear, and the research content and objective cannot meet the new requirements of improving the quality and efficiency and green development of China’s agriculture. In terms of research objectives, existing studies mostly focus on major food crops and mostly on a few varieties. However, the impact of warming on non-food crops is also significant, and there are significant differences between varieties of the same crop type. Studies on limited crop types and single varieties can hardly meet the innovative needs of adaptive technologies and coping strategies. With regard to research methods and means, especially field empirical research, most of the studies consider a single factor, and some involve two factors. It is urgent to establish multifactor comprehensive field facilities and corresponding comprehensive models to improve research methods that simulate the real climate system.

### Improving Methods and Techniques for Climate Change Impact Assessment

Finally, in terms of rice production technologies and models to cope with climate warming, we still focus on strategies with insufficient system integration of key technologies and inadequate adaptability and practicability of coping technologies. To reduce the impact of climate warming on rice supply and food security, rice production should consider multiple aspects, including how to improve the adaptive capacity of rice production systems. At the same time, it should also include how to promote coordination between soil organic carbon sequestration and greenhouse gas (GHG) emissions reduction in paddy fields, especially CH_4_ emission reduction, to contribute to mitigating climate warming and creating climate-smart agriculture (FAO, 2019).

### Focus on the Systematic Assessment of the Impact of Climate Change on Rice Production

Based on existing research, the author of climate smart rice technology system construction put forward the following suggestions: first, to strengthen the construction of the early warning and forecasting ability of climate change, high standard ecological field development, enrich varietal breeding and the rice planting technology supporting varietal creation, enhance paddy ecosystem comprehensive ability to adapt to climate warming, achieve high and stable yield of rice of high quality for security; The current change in climate is not a uniform process of warming, frequent extreme weather disaster events have increased the risk to agricultural production, resulting in the need to set up an extreme weather early warning and forecasting system, such as for heavy rain, seasonal drought, extreme temperature, and other natural disasters, which will reduce the risk of disaster. At the same time the government should take the lead in building modern farmland, paddy production facilities, breeding of high-yield and stress-resistant rice varieties, supporting modern rice farming techniques, and popularizing eco-friendly rice production methods (Long, 2016). Second, we should strengthen the optimization and distribution of rice production systems and paddy field ecosystems, promote the extension of the industrial chain, improve the quality and efficiency of agricultural products, achieve agricultural efficiency, and increase farmers’ income. For example, with the increase in heat resources and the demand for high-quality rice, rice production can be moderately expanded in northeast China, and high-quality rice varieties with long growth cycles can be promoted to produce high-quality rice. Meanwhile, rice industry clusters can be built to build brands and increase economic benefits (Antle John, 1995; Kassam et al., 2015; Chen et al., 2017a). Third, attention should be given to land use planning in rice-growing areas, the improvement of soil organic matter in paddy fields and the increase or reduction of agricultural chemicals to improve the storage capacity of organic carbon in agricultural systems, especially farmland soil, and reduction of GHG emissions from agricultural sources as much as possible. According to the actual circumstances of the rice planting areas, to carry out appropriate supporting cultivation measures, such as green manure cropping winter cover and protective lime amendment measures can be implemented. Additionally, measures such as the promotion of returned straw and intermittent irrigation, and the promotion of soil testing formulas and precise fertilization and the development of carbon reduction emissions of rice planting patterns can be expanded (Jiang R. et al., 2017; Jiang et al., 2018, 2019; Song et al., 2019; Qian et al., 2022). The author thinks intelligent rice technology should include three modules, namely, the rice productivity technology (adaptive cultivation technology), soil organic carbon sequestration technology and paddy GHG emissions reduction technology. Through technology integration, innovation and, mode of implementation food security will be safeguarded, rice farmers livelihoods will be improved and, climate warming will slow resulting in a mutually beneficial outcome by promoting sustainable development of rice industry.

## Author Contributions

DW, SF, and SS designed the work. MA gathered the literature related to the manuscript. HFA, AB, AM, NA, SSA, HA, and SS participated and analyzed the data from the experiments. SH, RS, SA, and SF reviewed and polished the manuscript. All authors contributed to the article and approved the submitted version.

## Conflict of Interest

The authors declare that the research was conducted in the absence of any commercial or financial relationships that could be construed as a potential conflict of interest.

## Publisher’s Note

All claims expressed in this article are solely those of the authors and do not necessarily represent those of their affiliated organizations, or those of the publisher, the editors and the reviewers. Any product that may be evaluated in this article, or claim that may be made by its manufacturer, is not guaranteed or endorsed by the publisher.
